# Blood-brain barrier penetration and tissue distribution of the selective membrane progesterone receptor α (mPRα/PAQR7) agonist Org OD 02-0

**DOI:** 10.3389/fendo.2026.1870020

**Published:** 2026-08-19

**Authors:** László Ákos Kovács, János Schmidt, Golgol Abolfazl, Zsolt Pirger, István Fodor

**Affiliations:** 1 Department of Anatomy, Medical School, University of Pécs, Pécs, Hungary; 2 Research Group for Mood Disorders, Centre for Neuroscience, Medical School, University of Pécs, Pécs, Hungary; 3 Institute of Biochemistry and Medical Chemistry, Medical School, University of Pécs, Pécs, Hungary; 4 Ecophysiological and Environmental Toxicological Research Group, HUN-REN Balaton Limnological Research Institute, Tihany, Hungary

**Keywords:** blood-brain barrier, membrane progesterone receptor α, Org OD 02-0, Parkinson’s disease, tissue distribution, Wistar rat

## Abstract

Parkinson’s disease (PD) remains a major neurodegenerative disorder with limited therapeutic options. Several *in vivo* animal studies using different experimental PD models have shown that progesterone (P4) may exert neuroprotective effects; however, the exact cellular and molecular mechanisms, including the receptors mediating these actions of P4, have not been investigated. A recent study from the laboratory of Peter Thomas demonstrated that the selective membrane progesterone receptor α (mPRα/PAQR7) agonist Org OD 02-0 (10-ethenyl-19-norprogesterone) exhibits neuroprotective effects in a human PD cell model, identifying mPRα as a promising target for the development of therapeutic strategies for PD prevention and management. However, the blood-brain barrier (BBB) penetration and *in vivo* pharmacokinetics of Org OD 02–0 have not yet been examined. Therefore, the aim of the present pilot study was to investigate the BBB penetration and tissue distribution of Org OD 02–0 following systemic administration in rats. To achieve this, we first developed a sensitive high-performance liquid chromatography-coupled mass spectrometry method for the detection and quantification of Org OD 02–0 in rat tissues. Using this method, we investigated the distribution of Org OD 02–0 in male Wistar rats following subcutaneous administration (2.6 mg/kg). Org OD 02–0 was detectable in all examined tissues, showing higher concentrations in the brain (frontal lobe) and kidney and lower levels in plasma and liver at 1, 3, 6, and 12 h post-injection. The concentration data suggested differential tissue distribution and potential preferential accumulation in the kidneys. Importantly, our results demonstrate, for the first time, that Org OD 02–0 crosses the BBB and reaches pharmacologically relevant concentrations in the central nervous system following systemic administration, supporting its potential as a candidate for PD therapy. Based on these data, *in vivo* studies in rat PD models are warranted to further evaluate the therapeutic efficacy of Org OD 02-0.

## 1 Introduction

Parkinson’s disease (PD) is one of the neurodegenerative diseases, affecting both motor (e.g., tremors, bradykinesia) and non-motor (e.g., mood swings) functions (1). As of 2024, PD is the second most common neurodegenerative disease and the fastest-growing in terms of total number of cases (2). Although research on PD has advanced significantly, there are still many open questions and critical issues to be resolved, including key aspects of its etiology, pathogenesis, and effective treatments (1, 3). PD is a spectrum disorder, whereas current pharmacological and surgical treatments primarily alleviate the motor symptoms of the disease (4, 5). However, PD is also characterized by a wide range of non-motor symptoms (6). Most currently approved pharmacological therapies target the dopaminergic system and include dopamine replacement therapies, dopamine receptor agonists, monoamine oxidase-B inhibitors, and catechol-O-methyltransferase inhibitors. Although these treatments effectively improve motor symptoms, they are frequently associated with adverse effects, including dyskinesia, nausea, hallucinations, orthostatic hypotension, and diarrhea (5), and are generally less effective in treating the non-motor symptoms of PD (7). The adverse effects of currently available therapies, together with their limited efficacy in treating non-motor symptoms, highlight the need for the development of novel therapeutic approaches targeting alternative disease mechanisms (4, 7).

The higher incidence of PD in men, together with evidence of delayed symptom onset in women, has led to the hypothesis that female sex hormones exert neuroprotective effects. Many *in vivo* studies using the 1-methyl-4-phenyl-1,2,3,6-tetrahydropyridine (MPTP)-induced PD model have demonstrated the neuroprotective actions of progesterone (P4) when administered before exposure (8–11), and have also suggested that it may reduce neurotoxicity if given shortly after the insult (12). However, the exact cellular and molecular mechanisms, including the receptors mediating these neuroprotective actions of P4, have not been revealed.

Since 2010, studies have investigated the potential neuroprotective roles of membrane progesterone receptors (mPRs). mPRs are members of the progestin and adipoQ receptor family, exert their actions in both reproductive and non-reproductive tissues, and have five isoforms: mPRα, mPRβ, mPRγ, mPRδ, and mPRϵ [reviewed by (13, 14)]. mPRs have been reported to mediate P4 neuroprotective actions in different neuronal cell line models (15–17). Highlighting, the synthetic P4 derivative Org OD 02-0 (10-ethenyl-19-norprogesterone), which is a selective mPR agonist (18), displayed neuroprotective activity in SH-SY5Y cells, a neuroblastoma cell line widely used in PD research (19), in a model of cell starvation (15). Moreover, a recent study from the laboratory of Peter Thomas demonstrated that Org OD 02–0 exhibits neuroprotective effects in SH-SY5Y cells using two established pharmacological treatments for cellular PD models, 6-hydroxydopamine and 1-methyl-4-phenylpyridinium (the active metabolite of MPTP), by binding to mPRα and activating the PI3K-AKT and MAP kinase signaling pathways (20). These findings highlighted mPRα as a promising target for the development of therapeutic strategies for PD prevention or management. However, a key unanswered question is whether these promising *in vitro* effects can be translated into an *in vivo* context, as the blood-brain barrier (BBB) penetration and pharmacokinetics of Org OD 02–0 have not yet been investigated.

In the present pilot study, we developed a sensitive high-performance liquid chromatography-coupled mass spectrometry (HPLC-MS) method for the detection and quantification of Org OD 02–0 in rat tissues. Using this method, we investigated the distribution of Org OD 02–0 in male Wistar rats following subcutaneous administration (2.6 mg/kg). Our results demonstrate that Org OD 02–0 crosses the BBB and support it as a potential candidate for PD therapy.

## 2 Materials and methods

### 2.1 Chemicals

**Figure 1:**
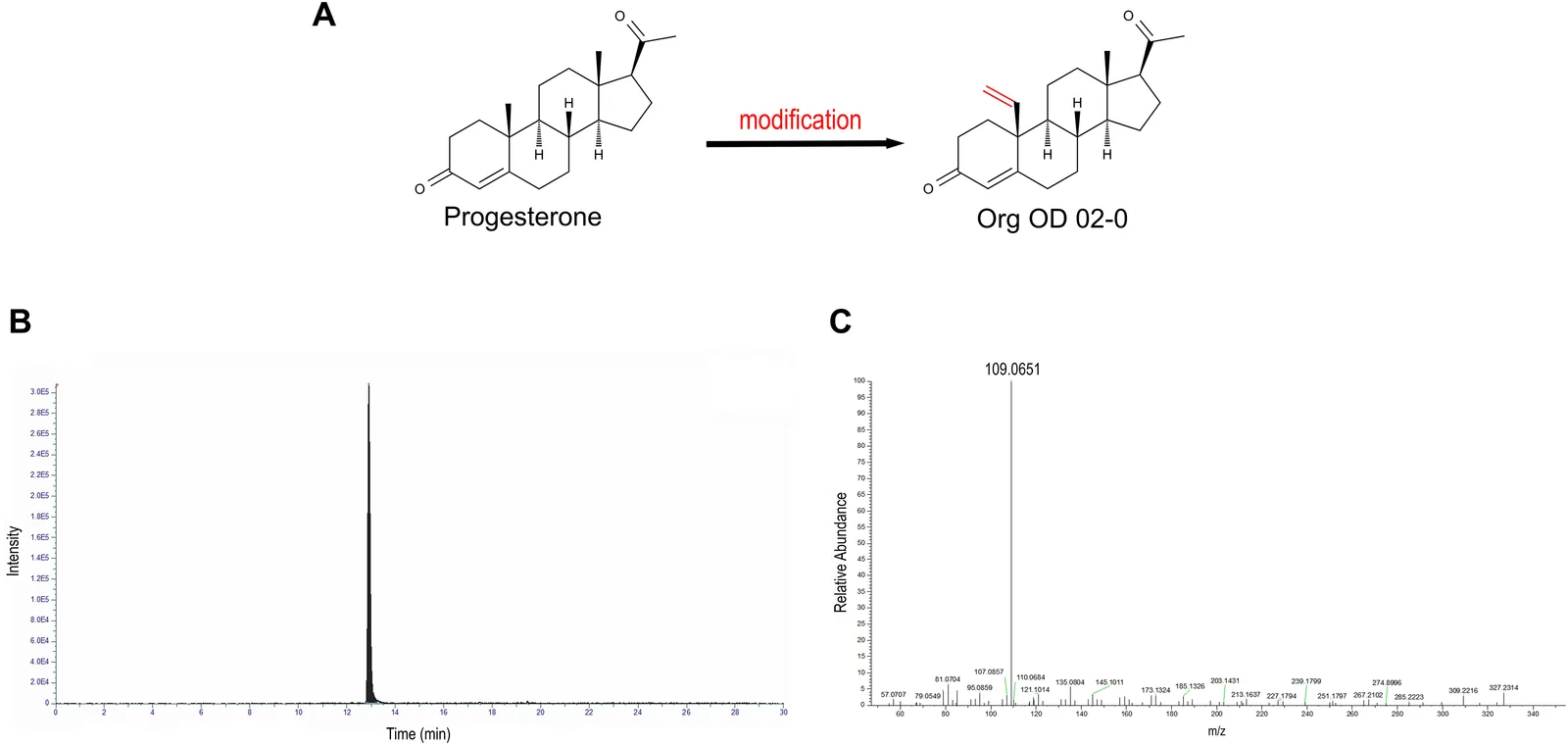
Chemical structure and analytical characterization of Org OD 02-0. **(A)** Chemical structures of progesterone and its analog, 10-ethenyl-19-norprogesterone (Org OD 02-0). The modification (indicated in red) results in a selective membrane progesterone receptor (mPRα) agonist. **(B)** Representative HPLC-MS/MS chromatogram acquired using parallel reaction monitoring (PRM) of a frontal lobe tissue extract obtained 3 h post-administration. Org OD 02–0 shows a distinct peak at a retention time (RT) of 12.88 min. **(C)** MS/MS fragmentation spectrum of Org OD 02-0, showing the characteristic fragmentation from the precursor ion 327.23 [M+H]^+^ to the dominant product ion 109.06, which was used for targeted quantification.

The specific mPR agonist Org OD 02-0 (**Figure 1A**) (#2085) was purchased from Axon Medchem (Groningen, Netherlands).

### 2.2 In silico evaluation of the BBB permeability of Org OD 02-0

BBB permeability of Org OD 02–0 was first evaluated using several widely applied *in silico* tools, including SwissADME (21), admetSAR (22), and pkCSM (23). For all predictions, the molecular structure of Org OD 02–0 was encoded in SMILES (Simplified Molecular Input Line Entry System) format and used as input.

### 2.3 Ethical approval

The applied protocols and procedures were approved by the Animal Welfare Committee of the University of Pécs and the National Scientific Ethical Committee on Animal Experimentation of Hungary. Licenses were granted by the Government Office of Baranya County (BA02/2000-14/2025). They were also in accordance with the directives of the European Union (86/609/EEC, Directive 2010/63/EU) and the rules of the Hungarian Government (40/2013).II.14.) on the protection of animals used for scientific purposes.

### 2.4 Experimental animals and treatments for pharmacokinetics

Animals were housed in groups of two or three rats per standard polycarbonate cage (40 × 25 × 20 cm) at a neutral temperature (24 °C) in a humidity-controlled environment. The rooms of the animal facility were maintained on a 12 h light/dark cycle, with the light phase starting at 6:00 a.m. Rats were provided with standard rodent chow and tap water *ad libitum*. For this study, twenty-four male Wistar rats were selected from our laboratory-bred stock (Department of Anatomy, Medical School, University of Pécs). The body weights ranged from 320 to 412 g per animal, and a total of 24 animals were included in the experiment: 5 animals in the control group, 5 animals in each of the 1h, 3h, and 6h groups, and 4 animals in the 12h group (see **Table 1**).

**Table 1:** Number and body weight of the experimental animals.

Experimental animals		
Animal group	Number of animals	Body weight
Control group	5	355±1.63
1h group	5	383±3.72
3h group	5	381±4.12
6h group	5	375±2.67
12h group	4	341±3.74

**Figure 2:**
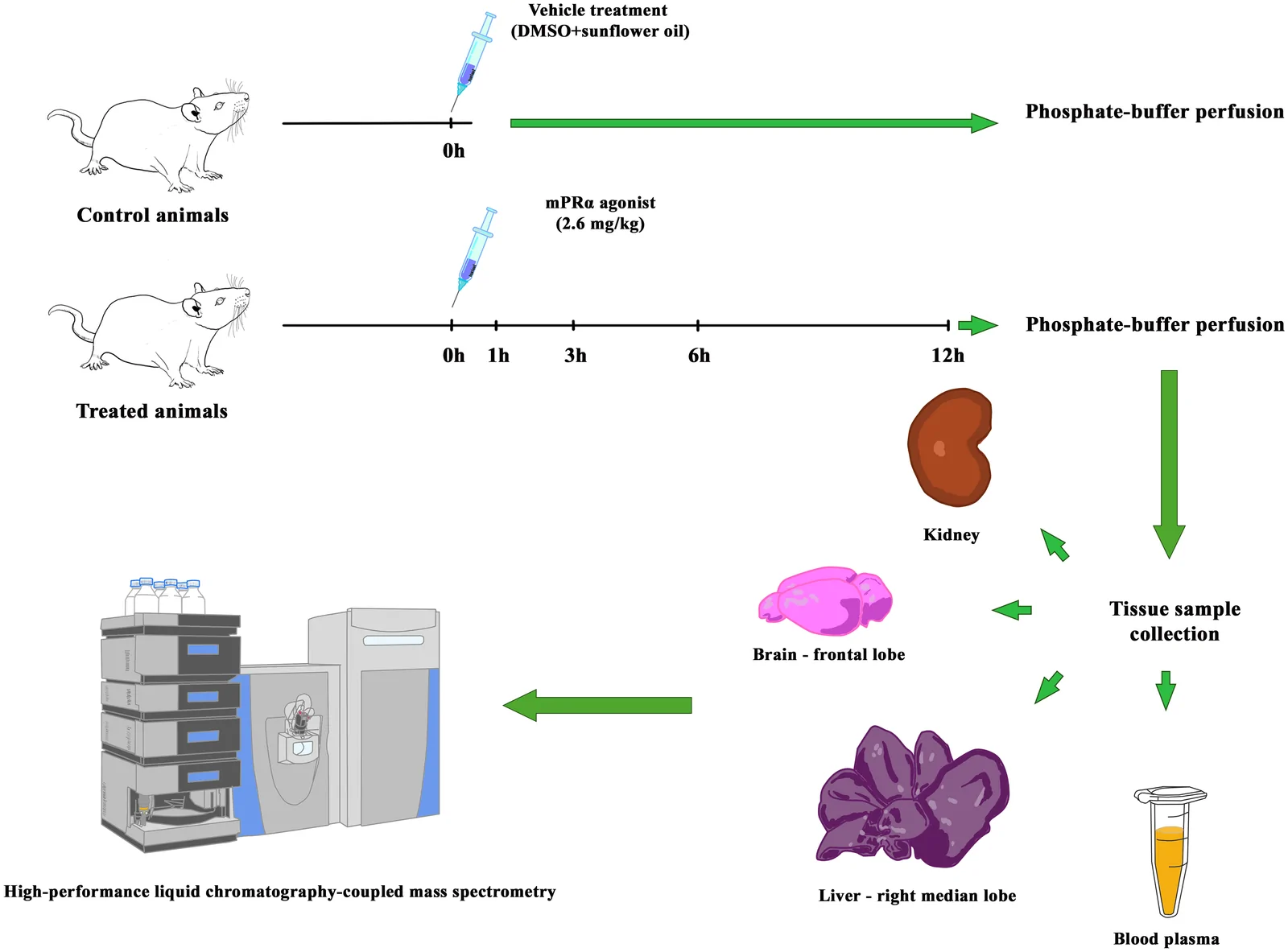
Experimental design and tissue collection workflow. Control animals received vehicle treatment (DMSO and sunflower oil) and were immediately anesthetized and perfused with phosphate-buffered saline (0h). Treated animals received the specific mPRα agonist Org OD-02 (2.6 mg/kg) and were anesthetized and perfused with phosphate-buffered saline at different time points post-treatment (1, 3, 6, and 12 h). Following perfusion, tissue samples from the brain (frontal lobe), kidney, liver (right median lobe), and blood plasma were collected. All samples were subsequently processed and analyzed using high-performance liquid chromatography coupled with mass spectrometry to determine the tissue concentrations of Org OD 02-0.

To test the metabolism and clearance of Org OD 02-0, we selected five animals as zero hour (controls) that received vehicle only (dimethyl sulfoxide [DMSO] in sunflower oil). Other nineteen animals were treated with Org OD 02-0 (dissolved in DMSO and diluted with sunflower oil as a vehicle) (**Figure 2**). Following treatment, the animals were returned to their home cages until anesthesia.

Org OD 02–0 is a lipophilic molecule. Based on our previous protocols for parkinsonian models (24, 25), we tested DMSO as a solvent. Limited data are available on the effective dose of P4 in rodent models of Parkinson’s disease (26). Collectively, these previous studies tested the 4–8 mg/kg dose range, which seems promising in rat models. Another critical aspect/limitation of dose selection is identifying an optimal/effective dose while minimizing side effects, particularly regarding future repeated administration (e.g., in parkinsonian models). The most critical step was the mixture of solutions: due to the known neurotoxicity of DMSO (27), efforts were made to improve solubility while limiting exposure. The compound was first dissolved in DMSO using vortex mixing, warming, and sonication (30 min). Following dissolution, sunflower oil was added as a vehicle, and the mixture was further sonicated and warmed for an additional 10 min. Due to these technical considerations (solubility, relatively large body weights of animals), we chose the ~2.5 mg/kg dose of Org OD 02–0 to achieve the most optimal result of this pilot study. Therefore, the treated animals received a 2.6 mg/kg dose of Org OD 02–0 prepared in 5 µL DMSO and 250 µL sunflower oil. Injection was administered subcutaneously to test the distribution of Org OD 02–0 from this compartment. Subcutaneous administration prolongs the absorption of steroids and, based on our experience with parkinsonian models, is a safer method for the animals (24, 28). The control animals received 5 µL DMSO mixed with 250 µL of sunflower oil. The animals were perfused at different time points after Org OD 02–0 treatment: 1, 3, 6, and 12 h after the injection (**Table 1**; **Figure 2**). Animals in the control group were deeply anesthetized with an overdose of intraperitoneal urethane (2.4 g/kg) immediately after vehicle administration (29, 30). Animals in the treated groups were anesthetized in the same manner 1, 3, 6, or 12h after Org OD 02–0 administration. The Org OD 02–0 was administered at 8:00, and the time of anesthesia and sacrifice was at 9:00 (1h), 11:00 (3h), 14:00 (6h), and 20:00 (12h). Following confirmation of deep anesthesia, blood samples were collected after transcardial perfusion, and the animals were euthanized by decapitation.

### 2.5 Sample collection

Four tissue samples (plasma, brain, liver, and kidney) were collected for HPLC-MS analysis (**Figure 2**). After successful anesthesia, the chest cavity was rapidly opened. A small incision was made in the left ventricle, and a blood sample for Org OD 02–0 measurement (see later) was collected. After blood sampling, a cannula was inserted into the ascending aorta and an incision was made in the right atrium. The rats were transcardially perfused with 400 mL 0.1 M phosphate-buffered saline (PBS; pH = 7.4) for 20 min. Finally, the animals were decapitated, and their brains were gently removed and frozen on dry ice for further analysis. From the collected brain samples, the frontal lobe was dissected and used for HPLC-MS analysis. This region was selected because it can be readily dissected and prepared for analysis, and it lacks the discontinuous BBB capillaries present in the diencephalon and brainstem. The abdominal wall of the animals was opened, and the entire left kidney and right median lobe of the liver were removed and frozen on dry ice. Frozen samples (frontal lobe, liver, and kidney) were stored at -80 °C until further analysis. For the determination of blood plasma Org OD 02–0 concentration, approximately 1 mL of left ventricular blood was collected just before perfusion into pre-chilled plastic tubes containing 150 μL of 8 m/m% EDTA solution (30). After centrifugation at 3500 rpm for 5 min at 4 °C, the plasma fraction was isolated, and 100 μL samples were stored at -80 °C until further analysis.

### 2.6 Sample preparation

Tissue (100 ± 2 mg) and plasma (100 µL) samples were transferred to new 1.5 mL Eppendorf tubes. In the first step, 70 µL of ultrapure water was added to the samples, which were then manually homogenized using plastic pestles. Following this, homogenization was continued on ice using a VCX 130 ultrasonic homogenizer (Sonics & Materials, Inc, USA). Each sample was treated with a microtip probe at 50% amplitude for 15 seconds; this process was repeated three times with a short cooling interval between bursts. After homogenization, 1 mL of methanol containing 0.1% formic acid was added to each sample, and the samples were vortex mixed for 15 minutes. Then, the samples were centrifuged at 20,000 × *g* for 20 minutes at room temperature using an Eppendorf 5430 R centrifuge (Eppendorf GmbH, Germany). After centrifugation, the supernatants were carefully collected, and the extraction procedure was repeated on the pellet. The solvents of the extracts were evaporated completely using an Eppendorf Concentrator Plus system. The evaporated samples were reconstituted in 500 µL of methanol containing 0.1% formic acid, and the extracts from the two extraction steps were pooled. Subsequently, the Eppendorf tubes were centrifuged again at 20,000 × *g* for 15 minutes at 7 °C. Finally, 100 µL of each extract was transferred into 1.5 mL autosampler glass vials for further analysis.

### 2.7 HPLC-MS analysis

LC-MS analysis was performed using a Q Exactive Orbitrap mass spectrometer (Thermo Fisher Scientific, USA) coupled to a Thermo Ultimate 3000 UPLC™ system. Chromatographic separation was performed on a Luna Omega PS C18 reversed-phase column (1.6 µm, 2.1 mm × 150 mm i.d.) (Phenomenex, USA). The mobile phase consisted of two solvents: solvent A was water/formic acid (99.9:0.1, v/v), and solvent B was methanol/isopropanol/formic acid (60:40:0.1, v/v/v). Both eluents contained 10 mM ammonium formate. The flow rate was 160 µL min^−1^, the column temperature was maintained at 45 °C, the injection volume was 5 µL, and the autosampler temperature was 7 °C. The gradient program included the following steps: from 0.0 to 10.0 minutes, the composition of solvent B increased from 40.0% to 80.0%; from 10.0 to 12.0 minutes, it increased from 80.0% to 100.0%; from 12.0 to 22.0 minutes, it was held at 100.0%; from 22.0 to 24.0 minutes, it decreased from 100.0% to 40.0%; and from 24.0 to 30.0 minutes, it was held at 40.0% for re-equilibration. The mass spectrometer was operated in positive ion mode using parallel reaction monitoring (PRM) for data acquisition. HESI source conditions were as follows: capillary temperature 250 °C, S-Lens RF level 60, spray voltage 3.5 kV, sheath gas flow 35 (arbitrary units), auxiliary gas flow 8 (arbitrary units). The resolution was set to 35,000, and the quadrupole isolation window was set to 2 Da. For molecular ion fragmentation, a normalized collision energy (NCE) of 29 was used. The target compound was quantified based on its specific transition of *m/z* 327.23 [M+H]^+^ → 109.06. Quantification was carried out using matrix-matched calibration curves generated in blank frontal lobe, kidney, liver, and plasma matrices spiked with Org OD 02–0 at 0.5, 1, 5, 10, and 50 ng/mL. Data acquisition and processing were performed using Xcalibur 4.2 software (Thermo Fisher Scientific).

### 2.8 Statistical analysis

**Figure 3:**
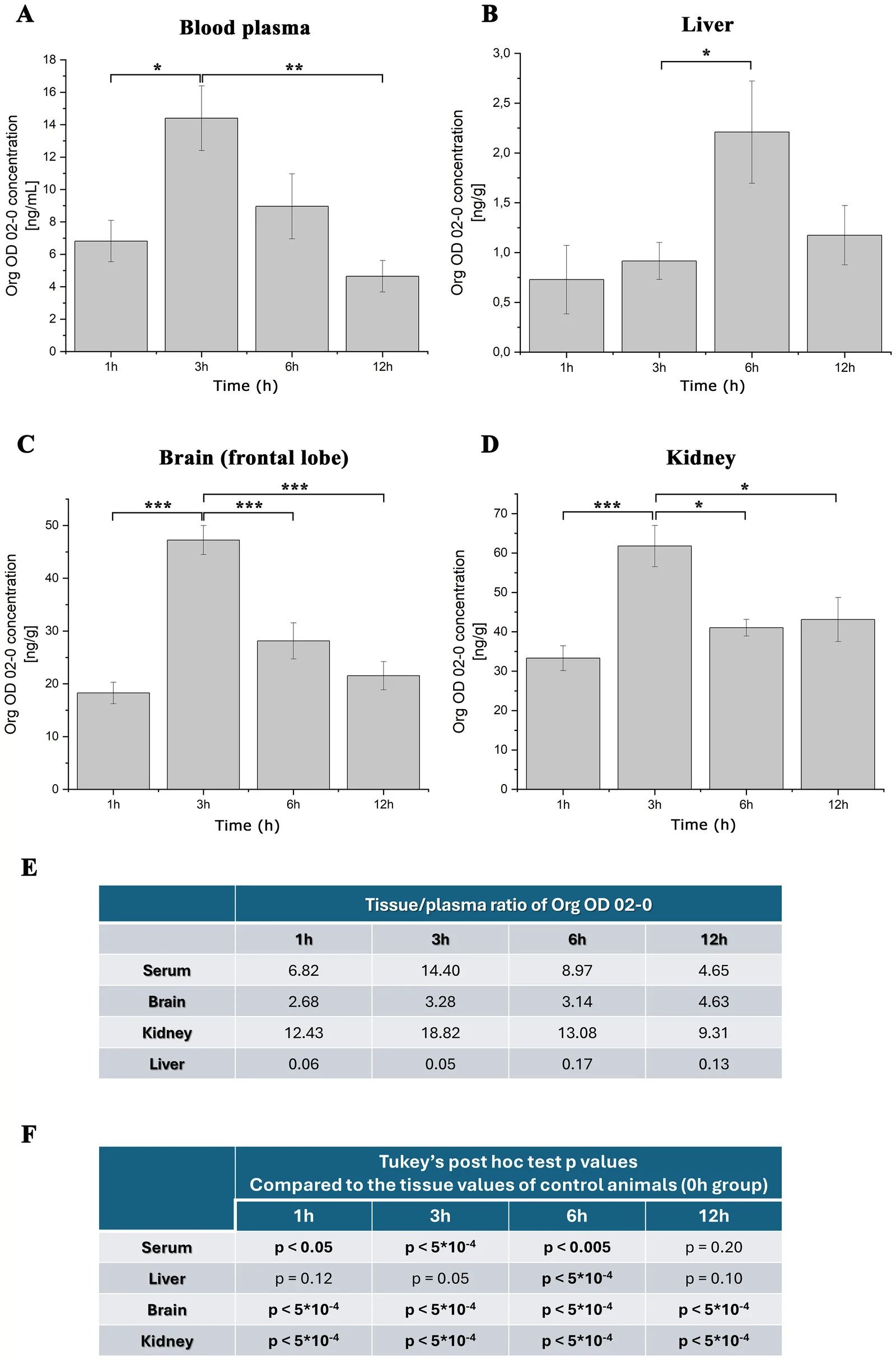
Concentrations of Org OD 02–0 in blood plasma **(A)**, liver **(B)**, frontal lobe **(C)**, and kidney **(D)** following subcutaneous administration. Samples were collected 1, 3, 6, and 12h after treatment of Org OD 02-0. Data are shown as mean ± SEM. Statistical significance between indicated groups was determined using one-way ANOVA followed by Tukey’s *post hoc* test (*p < 0.05, **p < 0.01, ***p < 0.001). **(E)** Tissue-to-plasma concentration ratios at each time point. **(F)** Tukey’s *post hoc* test p-values for comparisons between treated groups and the control group (0 h group), which are not shown in panels **(A–D)**.

Statistical analysis was made with the OriginPro 2018 software (OriginLab Corp., USA) and R v4.2.0 (R Core Team, 2025) programming environment. The normality of the datasets was investigated using the Shapiro-Wilk test and the homogeneity of variances between groups was investigated using the Levene-test. This analysis was followed by ANOVA with Tukey *post hoc* tests to identify significant differences between control (0h) and treated (1h, 3h, 6h, 12h) groups at a given time point (**Figure 3F**). In addition, we compared the time groups (1h, 3h, 6h, 12h) to detect the time dependent changes (**Figures 3A–D**).

## 3 Results and discussion

### 3.1 Analytical method for measuring Org OD 02–0 in rat tissues

A sensitive LC-MS method was successfully developed for the quantification of Org OD 02–0 in different rat tissues, including brain (frontal lobe), kidney, liver, and plasma. To the best of our knowledge, this is the first reported analytical method enabling the detection and quantification of Org OD 02–0 from tissue samples, as previous studies have mainly dealt with pharmacological assays without direct MS validation. The chromatographic separation and MS/MS spectrum of Org OD 02–0 are presented in **Figures 1B, C**. Steroids and steroid-like compounds are known to exhibit strong binding to lipids and plasma proteins, which can significantly affect extraction efficiency and analytical recovery - the sample preparation protocol was specifically optimized to account for this. The method demonstrated excellent linearity (r² = 0.998-0.999 across matrices), as well as good accuracy (89-111%) and precision (CV <8%). Extraction recoveries ranged between 62% and 85% across different matrices, and matrix effect was compensated through matrix-matched calibration, ensuring reliable quantification. The LOD was 0.1 ng/mL, whereas the LOQ was 0.5 ng/mL in all matrices. A limitation of the method is the absence of an isotopically labeled internal standard, which may affect the correction of matrix effects, extraction variability, and instrumental fluctuations. Although the use of matrix-matched calibration partially compensates for these factors, some degree of analytical variability remains inherent to the method. Hence, the inclusion of a suitable isotopically labeled internal standard in future studies would further improve quantitative robustness and accuracy. Overall, the developed analytical method provides a reliable and reproducible tool for the analysis of Org OD 02–0 in different tissues and can serve as a basis for future pharmacokinetic and mechanistic studies.

### 3.2 BBB penetration and tissue distribution of Org OD 02-0

BBB permeability of Org OD 02–0 was first evaluated using several widely applied *in silico* tools, including SwissADME, admetSAR, and pkCSM. Notably, all three methods consistently indicated that the compound is likely to cross the BBB and reach the central nervous system (**Supplementary Figure 1**), indicating its suitability for further *in vivo* studies.

**Figure 4:**
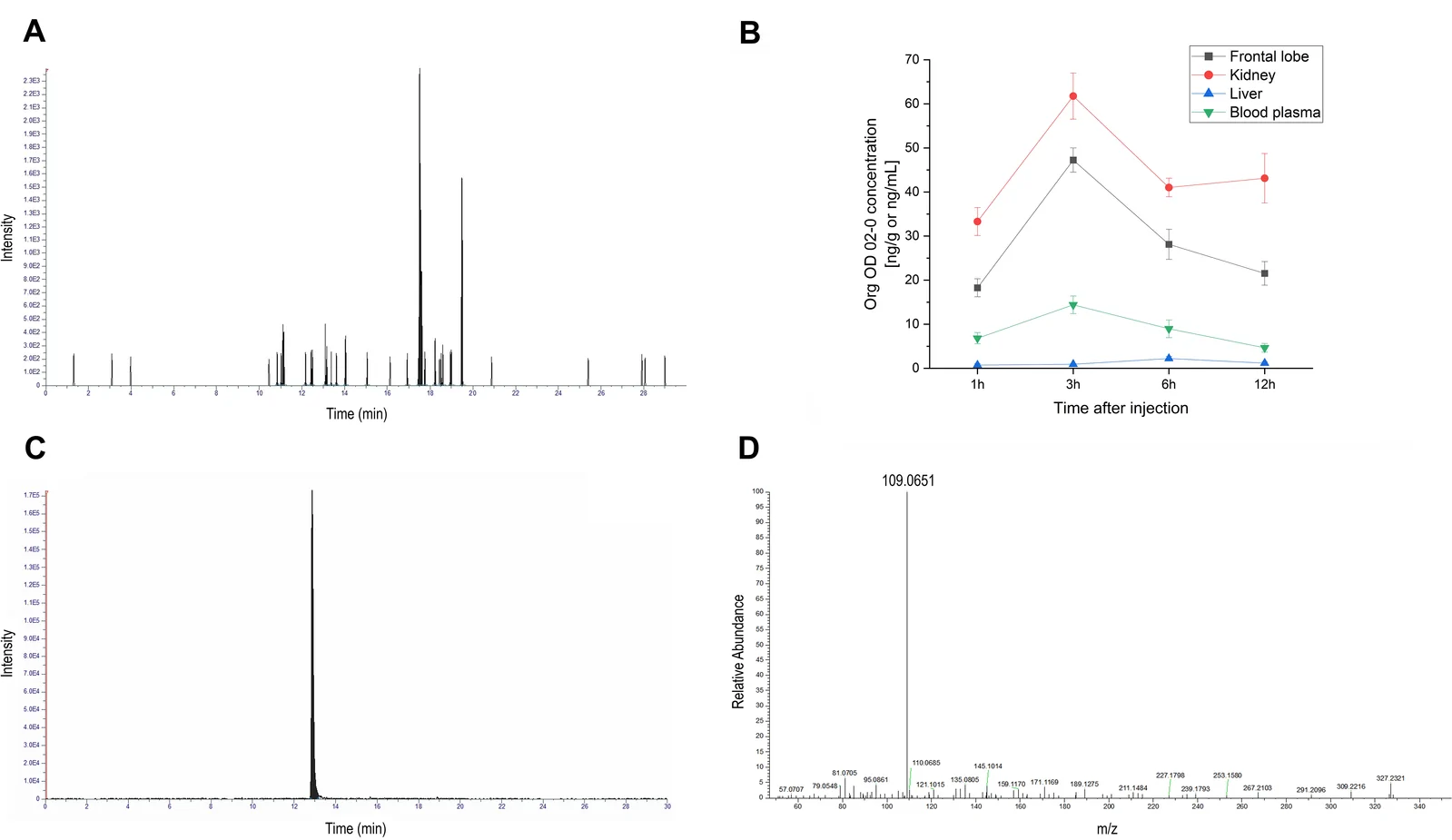
Tissue distribution of Org OD 02–0 following systemic administration. **(A)** Representative HPLC-MS/MS chromatogram of a control frontal lobe sample. The trace confirms the absence of interfering endogenous peaks at the retention time of the target compound, demonstrating the selectivity of the analytical method. **(B)** Time-dependent concentration profiles of Org OD 02–0 in various tissues. Data represent the mean concentration (ng/g for tissues or ng/mL for plasma) ± SEM in the frontal lobe, kidney, liver, and plasma at 1, 3, 6, and 12 h post-injection. Peak concentrations were observed at the 3-hour time point across all compartments, with the highest accumulation detected in the kidney and the frontal lobe. **(C)** Representative HPLC-MS/MS chromatogram of a frontal lobe sample collected 3 h post-administration. A robust and well-resolved peak is visible at RT 12.88 min, corresponding to the quantified Org OD 02-0. **(D)** MS/MS fragmentation spectrum of Org OD 02–0 from the biological matrix. The spectrum confirms the identity of the compound in the tissue extract, showing the characteristic precursor ion m/z 327.23 [M+H]^+^ and the dominant product ion m/z 109.06 used for quantification.

Org OD 02–0 was not detected in the control animals (0h) (**Figure 4A**) (**Supplementary Information**) but was detectable in all examined tissues at each investigated time point (1, 3, 6, and 12 h) following subcutaneous administration (**Figure 4B**; **Supplementary Information**), demonstrating a systemic distribution. According to ANOVA (F_1,4_ = 13.36, p < 5*10^-4^), blood plasma concentrations (**Figure 3F**) demonstrated a significant presence of Org OD 02–0 in the 1h (p < 0.05), 3h (p < 0.005), and 6h (p < 0.005) groups compared with the control animals. The tissue-to-plasma ratio ranged between 2.67 to 4.63 in the frontal lobe, 9.31 to 18.82 in the kidney, and 0.05 to 0.17 in the liver (**Figure 3E**). These results indicate marked accumulation of Org OD 02–0 in the brain and kidney, whereas only very low levels were detected in the liver.

It is well-known that intravenous or intraperitoneal administration of P4 and synthetic progestins results in higher initial concentrations in both plasma and liver, with peak levels occurring within minutes (31), followed by a rapid decline. In contrast, topical or subcutaneous administration leads to prolonged absorption times (32, 33) and more sustained plasma concentrations, which may explain the continuous detectability of Org OD 02–0 in plasma throughout the observation period. At 3h, plasma concentrations of Org OD 02–0 were significantly higher than those measured at 1h (p < 0.05), 6 h (p < 0.005), and 12 h (p < 0.005), according to Tukey’s *post hoc* test (**Figure 3A**). This peak at 3h may indicate the redistribution of Org OD 02-0. Highly lipophilic compounds such as steroids typically exhibit high tissue-to-plasma ratio (K_p_ > 1; **Figure 3E**), indicating accumulation in lipid rich tissues including adipose tissue and the brain. Therefore, the secondary plasma peak observed at 3h may result from the delayed release of Org OD 02–0 from these tissue reservoirs back into the circulation. While Org OD 02–0 exhibited a modest plasma peak at 3h after administration, no pronounced peak was observed in the liver, and liver concentrations of Org OD 02–0 remained extremely low throughout the study period (**Figure 4B**). One-way ANOVA revealed a significant effect of treatment on liver Org OD 02–0 concentrations (F_1,4_ = 6,37 p<0.05) (**Figure 3B**). However, comparisons with the control group showed a significant difference only in the 6 h group (p < 0.001; **Figure 3F**). Hepatic cells exhibit extremely rapid metabolism of P4 (34–36); therefore, the detected Org OD 02–0 likely represents a residual compound derived from the plasma and undergoing metabolism. In general, a large proportion of P4 is carried by plasma proteins (albumin and corticosteroid-binding globulin) (37). However, during HPLC-MS sample preparation, protein-bound steroids are typically released, allowing the measurement of total circulating steroid concentrations. Even if we believe in prolonged subcutaneous absorption, we cannot exclude the presence of a primary undetected peak concentration in the plasma or liver samples in earlier times (groups). Additional treatment groups with earlier and later sampling time points (e.g., 5, 10, and 30 min, and 24 h post-injection), as well as multiple-dose administration, would be required for a comprehensive pharmacokinetic characterization of Org OD 02-0 (38). Moreover, adipose tissue (e.g., the retroperitoneal fat pad) and skeletal muscle were not included in the tissue collection. Consequently, the present dataset does not allow the construction of a bi- or multicompartment pharmacokinetic model necessary to quantify tissue redistribution and estimate key pharmacokinetic parameters.

The highest concentrations were observed in the kidney (F_1,4_ = 34.81, p < 10^-6^) and the frontal lobe of the brain (F_1,4_ = 47.60 p < 5*10^-6^), peaking 3h post-injection (**Figure 4B**). In the kidney, Org OD 02–0 reached the maximum measured tissue concentration (61.76 ± 5.22 ng/g). This concentration was significantly higher than those detected in the 1h (p < 0.001), 6h (p < 0.05), and 12h (p < 0.05) groups (**Figure 3C**). Protein-bound sex steroids are filtered by the glomerulus (as part of renal excretion). This process allows megalin and cubulin receptors in the renal epithelial cells of the proximal tubules to reabsorb these molecules (39–41). In addition, anion transporters can transport steroid molecules such as estrogen sulfate (42), a phenomenon that may contribute to the renal accumulation of Org OD 02-0. In addition to steroid transporters, various enzymes in the kidneys participate in the metabolism of P4 and testosterone (43, 44). The low plasma levels and high renal concentrations of Org OD 02–0 suggest preferential accumulation in the kidney, where the unbound fraction may be released or metabolized within tubular cells.

Similar to the kidney, Org OD 02–0 concentrations in the frontal lobe peaked at 3h, reaching 47.24 ± 2.75 ng/g (**Figures 4B–D**). The concentration measured at 3h was significantly higher than those observed at all other time points (p < 0.001; **Figure 3D**). P4 is well-known to easily pass in large quantities throughout the BBB of rats (45, 46). The BBB is actively involved in the transport and metabolism of steroids, contributing to steroid accumulation in brain tissue (47). In male rats, P4 is detected at higher concentrations in the brain than in blood plasma (48). Overall, the free Org OD 02–0 passed through the BBB and accumulated in the frontal lobe, similar to P4. At first sight, the detected level of Org OD 02–0 in the frontal lobe appears low amount for a biologically active compound. However, a previous study reported that the average concentration of P4 in the cerebral cortex of male rats ranges from 1.5 to 7.0 ng/g (48). Moreover, brain samples from 25-day-old pubertal female rats contained 19–29 ng/g P4 at 53–55 h following ovulation induced by gonadotropin-releasing hormone treatment (46). This amount is comparable to human data, where the P4 concentration was similar in the cerebral cortex (49). Collectively, these P4 data suggest that the concentrations of Org OD 02–0 detected in the frontal lobe may be sufficient to produce neuronal effects and may also have the potential to induce neuroprotective effects in future preclinical disease models. Importantly, (20) used Org OD 02–0 at 100 nM (equivalent to 32.65 ng/mL), which was effective in cell culture models of PD. The presence of Org OD 02–0 in the frontal lobe at all time points provides the first direct evidence that this selective mPRα agonist effectively crosses the BBB *in vivo*. Although a decrease was observed after 3 h, the compound remained detectable even at the latest point, indicating its stability and persistence in the brain.

Taken together, our findings provide the first *in vivo* evidence that the selective mPRα agonist Org OD 02–0 effectively crosses the BBB and reaches pharmacologically relevant concentrations in the brain following systemic administration. Its favorable tissue distribution properties, including rapid distribution, central nervous system availability, and sustained tissue exposure, support the further preclinical development of Org OD 02–0 as a potential therapeutic candidate for PD. Although this pilot study has several limitations, particularly the absence of data (i.e. lack of multiple-dose administration, limited number of sampling points, and limited tissue sampling) required for a detailed bi- or multicompartment pharmacokinetic analysis of Org OD 02-0, it establishes an essential pharmacokinetic foundation for future studies. These studies should evaluate the neuroprotective efficacy, optimal dosing regimen, long-term safety, and pharmacodynamic mechanisms of Org OD 02–0 to define its therapeutic potential and possible clinical niche in experimental models of neurodegenerative disorders, particularly PD.

## Data Availability

The original contributions presented in the study are included in the article/**Supplementary Material**. Further inquiries can be directed to the corresponding author.
