# Supplementary material for: Blood-brain barrier penetration and tissue distribution of the selective membrane progesterone receptor α (mPRα/PAQR7) agonist Org OD 02-0
Source: Front Endocrinol (Lausanne). 2026 Aug 19;17:1870020. doi: 10.3389/fendo.2026.1870020 (PMC13533746; doi:10.3389/fendo.2026.1870020)
Supplement: Supplementary file 1 [file DataSheet1.docx]

**Blood-brain barrier penetration and tissue distribution of the selective membrane progesterone receptor α (mPRα/PAQR7) agonist Org OD 02-0**

László Ákos Kovács^1,2+^, János Schmidt^3+^, Golgol Abolfazl^1,2^, Zsolt Pirger^4^, István Fodor^4^*

^1^Department of Anatomy, Medical School, University of Pécs, 7624, Pécs, Hungary

^2^Research Group for Mood Disorders, Centre for Neuroscience, University Medical School, University of Pécs, 7624, Pécs, Hungary

^3^Institute of Biochemistry and Medical Chemistry, Medical School, University of Pécs, 7624, Pécs, Hungary

^4^Ecophysiological and Environmental Toxicological Research Group, HUN-REN Balaton Limnological Research Institute, 8237, Tihany, Hungary

*Corresponding author; E-mail: [fodor.istvan@blki.hu](mailto:fodor.istvan@blki.hu) (I. Fodor)

^+^Equally contributed authors

**Supplementary Figure 1**. SwissADME BOILED-Egg diagram showing the ADME properties of Org OD 02-0. The plot displays WLOGP values (y-axis) versus TPSA values (x-axis), where the white ellipse indicates high human intestinal absorption (HIA) probability, the yellow area shows blood-brain barrier (BBB) penetration compounds, and the grey region marks poor absorption or penetration. The Org OD 02-0 molecule (yellow, labeled) lies in the BBB region, suggesting favorable BBB penetration. Blue dots denote P-glycoprotein (PGP+) substrates, red ones PGP- non-substrates. Ref: (1)

**Representative chromatogram and MS/MS spectrum of rat tissues (kidney, liver, and blood plasma) derived from control (0h) and 3h post-injected animals**

*control – kidney*

*control – liver*

*control – plasma*

*3h - kidney*

*3h – liver*

*3h – plasma*

**References**

1.	Daina A, Michielin O, Zoete V. SwissADME: a free web tool to evaluate pharmacokinetics, drug-likeness and medicinal chemistry friendliness of small molecules. *Sci Rep* (2017) 7:42717. doi: 10.1038/srep42717
